# Body mass index distribution in rheumatoid arthritis: a collaborative analysis from three large German rheumatoid arthritis databases

**DOI:** 10.1186/s13075-016-1043-9

**Published:** 2016-06-23

**Authors:** Katinka Albrecht, Adrian Richter, Johanna Callhoff, Dörte Huscher, Georg Schett, Anja Strangfeld, Angela Zink

**Affiliations:** German Rheumatism Research Centre, Epidemiology Unit, Charitéplatz 1, 10117 Berlin, Germany; Department of Internal Medicine, University of Erlangen-Nuremberg, Erlangen, Germany; Department of Rheumatology and Clinical Immunology, Charité University Hospital, Berlin, Germany

**Keywords:** Body mass index, Epidemiology, Rheumatoid arthritis, Obesity

## Abstract

**Background:**

METARTHROS (Metabolic impact on joint and bone disease) is a nationwide German network to investigate the overlap between inflammatory and metabolic diseases. The objective of this study was to compare the body mass index (BMI) distribution in patients with early and established rheumatoid arthritis (RA) with data from the general population, and to evaluate the association of BMI with patient characteristics and clinical markers.

**Methods:**

The BMI distribution was examined with data collected at inclusion of patients in the early arthritis cohort CAPEA, the biologics register RABBIT, and the National database of the German Collaborative Arthritis Centers. A data source with a representative sample of the German population (German Ageing Survey) was used as a comparator. BMI categories of <18.5 kg/m^2^ (underweight), 18.5 to <25 kg/m^2^ (normal weight), 25 to <30 kg/m^2^ (overweight), and ≥30 kg/m^2^ (obese) were used. Patients were stratified by age and sex, and compared to controls from the German Ageing Survey. Associations between BMI and markers of disease activity were analysed with non-parametric tests and linear models.

**Results:**

Data from 1207 (CAPEA), 12,230 (RABBIT), and 3424 (National database) RA patients and 6202 population controls were evaluated. The mean age was 56, 56, 62, and 62 years, respectively, the mean disease duration was 13 weeks, 9.9 years, and 13.5 years, respectively, and the mean disease activity score (DAS28) was 5.1, 5.2, and 3.1, respectively. In all RA cohorts, obesity was more frequent (23.8 %, 23.4 %, 21.4 %, respectively) than in controls (18.2 %). This applied to all age groups <70 years, was independent of disease duration, and was more pronounced in females. In all cohorts, the age at RA onset was associated with BMI, being higher in overweight/obese patients compared to normal-weight patients. Current smoking was negatively associated with BMI. Linear analyses revealed increased erythrocyte sedimentation rate (ESR) values in underweight and obese females, and an increasing disparity between tender joint counts (TJCs) and swollen joint counts (SJCs) in higher BMI categories.

**Conclusions:**

Compared to the general population, a higher prevalence of obesity was observed in all RA cohorts. The dominance of obesity in females and the different behaviour of disease activity markers in relation to the BMI in females indicate that additional parameters need to be considered when analysing the impact of obesity on inflammation in RA.

**Electronic supplementary material:**

The online version of this article (doi:10.1186/s13075-016-1043-9) contains supplementary material, which is available to authorized users.

## Background

Obesity has become a common condition in prosperous countries and its global rise has lead to subsequent morbidities. This situation also applies to auto-immune diseases such as rheumatoid arthritis (RA). Adipose tissue is known to have immunomodulatory as well as pro-inflammatory properties. Obesity impacts the development and progression of RA at different stages of the disease [1, 2]. Positive associations between obesity and the risk of developing RA have been reported, dominating in females [1, 3, 4]. The body composition is already altered in patients with early RA with more fat and less lean mass, with or without an increase in the body mass index (BMI) [5]. Various studies describe the association between high BMI categories and poorer clinical outcomes, a lower chance for remission, and a higher probability of comorbidity but less radiographic joint damage [2, 6]. Furthermore, sex-specific and conflicting results have been reported concerning inflammatory markers [6]. Additional factors, such as age, lifestyle, social status, physical activity, or comorbid conditions, may also influence the association between body weight and inflammation. Thus, some of these interactions may explain divergent results on the association with inflammatory markers in previous reports. Until now, the role of obesity in the course of RA and its involved mechanisms remain only incompletely understood [6].

METARTHROS (Metabolic impact on joint and bone disease) is a nationwide collaborative German network to investigate the interaction between inflammatory and metabolic diseases. The focus of this study was to investigate the prevalence of underweight, overweight and obese individuals in three large RA databases compared to representative data from the general population. In addition, the association of BMI status with socio-demographic characteristics and markers of disease activity was analysed.

## Methods

### Data sources

Three RA databases were used for analysis. The ‘Course And Prognosis of Early Arthritis’ (CAPEA) inception cohort is a prospective multicentre, non-interventional, observational study investigating the prognostic value of early symptoms for the development of a chronic disease course in patients with early arthritis. Patients with symptom duration of less than 6 months were included between 2010 and 2013 in 89 German rheumatologic institutions and were followed for 24 months [7]. Patients were included consecutively. The data at inclusion were selected. Only patients with a clinical RA diagnosis at the last documented visit were included in the analysis.

The National Database (NDB) of the German Collaborative Arthritis Centres is an on-going prospective study that was established in 1993 as a long-term monitoring system for German rheumatology patients. The database contains annually updated clinical data and patient-reported outcomes for unselected outpatients with inflammatory rheumatic diseases [8]. For the present study, cross-sectional data from the year 2013 from 16 arthritis centres were used.

The German biologics register (RABBIT—Rheumatoide Arthritis Beobachtung der Biologika Therapie) is an ongoing prospective, observational cohort study on the long-term safety and effectiveness of biologic and synthetic disease-modifying anti-rheumatic drug (DMARD) treatment in RA patients. Patients are eligible for enrolment at the start of treatment with a biologic or synthetic DMARD after failure of at least one DMARD. The register began in 2001 and contains patient data from approximately 350 rheumatologists nationwide. The patients are observed for up to 10 years [9].

All data sources provide information on demographics, details on rheumatologic visits, including laboratory tests, joint counts, and additional health information. They represent patients under routine rheumatologic care in a population-based setting. Prior to enrolment, all patients give their informed consent. All databases received ethical approval by the Ethics Committee of the Charité University Medicine, Berlin.

Data from the German microcensus and the German Ageing Survey were available as reference data. We found no differences between both data sources regarding the distribution of BMI, stratified by age (in years) and sex. Due to access to anonymized data on the individual person level, we selected the German Ageing Survey (DEAS) which has been provided by the Research Data Centre (FDZ-DEAS) of the German Centre of Gerontology (DZA) [10]. The DEAS is a longitudinal survey for the analysis of life situations and biographies of people in the second half of their lives. The data are based on nationally representative surveying (cross-sectional and longitudinal) of some more than 6000 participants from the age of 40 years onwards.

### Study design

We analysed the data from the inclusion visit in CAPEA and RABBIT and cross-sectional data from the NDB from the year 2013. The data were stratified by age, sex, and disease duration, and were compared separately for each RA cohort with data from the DEAS from the year 2008.

### Outcome and covariate assessment

The primary outcome was the BMI, which was calculated as the weight in kilograms divided by height in metres squared. It was classified into four groups according to the definition of the World Health Organization (WHO): underweight (<18.5 kg/m^2^, normal weight (18.5– < 25 kg/m^2^), overweight (25– < 30 kg/m^2^), and obese (≥30 kg/m^2^) [2].

Socio-demographics and clinical data were collected from the respective databases using the most recent (2013 for NDB) or baseline values (CAPEA, RABBIT). The following variables were investigated as covariates: age, sex, disease duration, C-reactive protein (CRP; mg/l), disease activity score of 28 joints (DAS28) and its components (erythrocyte sedimentation rate (ESR; mm/h), tender joint count (TJC), swollen joint count (SJC), and patient global assessment (NRS 0–10)). In addition, rheumatoid factor (RF), anti-citrullinated protein antibodies (CCP), functional capacity by Hannover Functional Assessment Questionnaire (FFbH; range 0–100, 100 representing full capacity) [11], smoking status (current/former/never), level of education (high/moderate/low), the number of comorbid conditions (reported by the rheumatologist), the number of current DMARDS, and the current glucocorticoid dose (mg/day) were documented.

### Statistical analysis

The baseline characteristics were compared between the RA cohorts and the population controls. The prevalence of obesity was compared by a sample proportion test.

The associations between BMI and socio-demographics were investigated using non-parametric tests. Regarding the association of BMI and markers of disease activity, generalized linear models (with normal or negative-binomial response) were applied. *P* < 0.05 was considered statistically significant.

## Results

### Study population

Data relating to 1033 patients with early arthritis (CAPEA), 12,230 patients with active RA after treatment failure (RABBIT), and 3424 unselected patients treated in rheumatology (NDB) were included in the present analysis and compared with data relating to 6202 population controls (DEAS). The patient characteristics are presented in Table 1.

**Table 1:** Patient characteristics of the arthritis cohorts and controls

	CAPEA	RABBIT	NDB	Reference (DEAS)
*N*	1033	12,230	3424	6202
Age (years), mean (SD)	56.6 (14.2)	56.2 (9.7)	61.5 (13.7)	61.5 (12.1)
Sex (female), n (%)	660 (63.9)	9328 (76.3)	2611 (76.3)	3072 (49.5)
Disease duration (weeks or years), mean (SD)	12.6 w (7.2)	9.9 y (9.1)	13.5 y (10.6)	NA
CRP (mg/l), mean (SD)	19.7 (30.3)	18.2 (26.6)	11.0 (5.2)	NA
DAS28, mean (SD)	5.1 (1.3)	5.2 (1.3)	3.1 (1.2)	NA
BMI (kg/m^2^), mean (SD)	27.0 (5.0)	26.7 (5.3)	26.5 (4.9)	26.6 (4.2)
< 18.5, n (%)	11 (1.1)	263 (2.2)	56 (1.6)	45 (0.8)
18.5 to <25, n (%)	372 (36.4)	4810 (39.3)	1416 (41.4)	2264 (37.5)
25 to <30, n (%)	396 (38.8)	4294 (35.1)	1219 (35.6)	2624 (43.5)
≥ 30, n (%)	243 (23.8)	2863 (23.4)	733 (21.4)	1099 (18.2)

*BMI* body mass index, *CRP* C-reactive protein, *DAS28* Disease Activity Score, *NA* not applicable, *SD* standard deviation, *w* weeks, *y* years

**Fig. 1:**
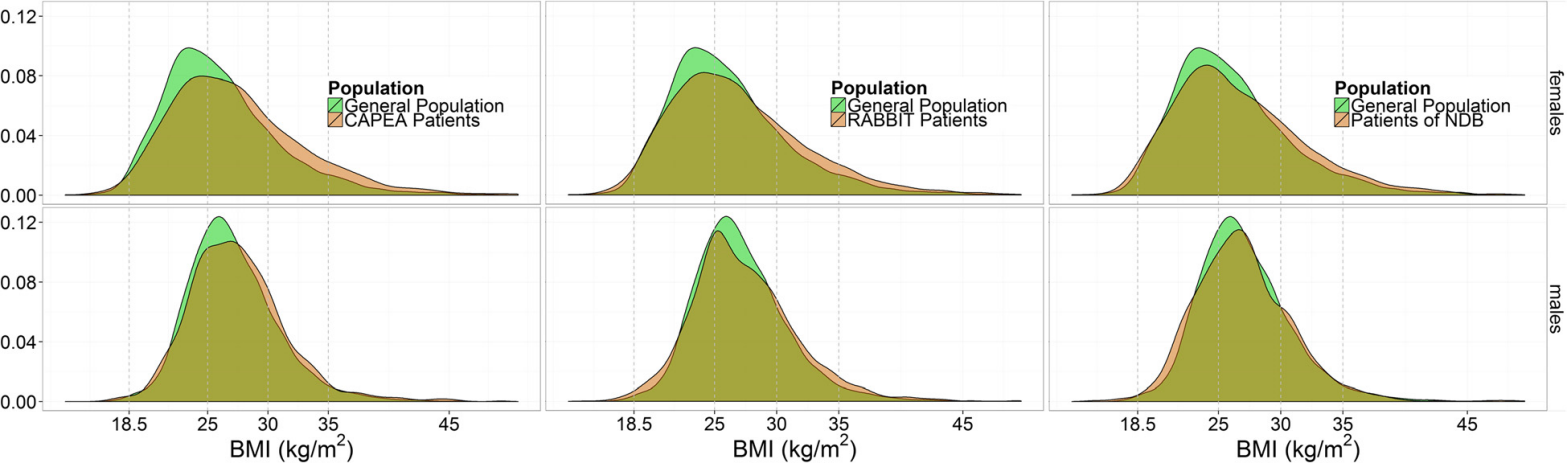
Body mass index distribution. The body mass index (*BMI*) was used to present the distribution of weight in the early arthritis cohort (*CAPEA*) and in patients with prevalent RA in the National Database (*NDB*) and in the biologics register (*RABBIT*) compared to the general population in the age group 40–79 years

The mean age in the NDB was comparable to the DEAS, whereas CAPEA and RABBIT patients were on average 4 years younger. The proportion of females was higher in the arthritis patients than in the controls. The mean DAS28 values of the arthritis cohorts reflected the varying disease activity at the onset (CAPEA), prior to a switch to a biologic or other csDMARD therapy after failure (RABBIT), and under current therapy (NDB). Taking the controls as reference, the strongest difference regarding BMI categories was found for obese patients, who were more frequently in all arthritis cohorts (all *P* < 0.01). Regarding the proportion of underweight patients, the difference was less prominent. Despite different disease duration and disease activity, all cohorts had a very similar BMI distribution (Fig. 1). The apparent differences to the normal population persisted when the comparison was additionally stratified by age decades.

### Distribution of BMI by age and sex

According to the age and sex differences in the cohorts and the reference group, the data were stratified, and each cohort was compared separately against the reference group. Table 2 shows the BMI distribution by sex and age groups in the arthritis cohorts and the reference data. The higher prevalence of obesity was most prominent in the age group <55 years. The prevalence of obesity remained independent of the disease duration.

**Table 2:** Distribution of the body mass index (BMI) by sex and age groups (%)

Cohort	BMI (kg/m^2^)	Females				Males			
		40– < 55	55– < 70	≥70	Total	40– < 55	55– < 70	≥70	Total
CAPEA	<18.5	1.0	1.0	2.9	1.4	0.9	0.8	0	0.6
	18.5 to <25	40.4	34.2	33.1	36.3	35.7	24.8	32.0	30.6
	25 to <30	32.7	34.6	42.5	35.8	43.8	52.0	45.0	47.2
	≥30	26.0	30.2	21.6	26.5	19.6	22.4	23.0	21.7
RABBIT	<18.5	2.6	1.7	2.0	2.1	0.5	0.7	0.5	0.6
	18.5 to <25	43.2	37.2	38.2	39.6	30.0	27.2	33.9	29.3
	25 to <30	29.8	35.3	36.8	33.6	45.5	46.7	49.9	46.8
	≥30	24.4	25.8	23	24.8	24	25.3	15.8	23.3
NDB	<18.5	2.0	1.2	2.4	1.8	1.0	0.3	0.7	0.6
	18.5 to <25	48.9	40.6	40.8	42.8	35.4	25.4	34.8	31.3
	25 to <30	26.0	34.3	36.5	33.0	43.9	49.8	48.4	47.8
	≥30	23.1	23.9	20.3	22.4	19.7	24.4	16.0	20.2
DEAS	<18.5	1.1	1.4	1.2	1.2	0.3	0.1	0.5	0.3
	18.5 to <25	54.9	43.4	36.7	45.7	34.4	26.9	28.6	29.8
	25 to <30	29.9	36.8	39.9	35.2	49.4	51.9	52.8	51.4
	≥30	14.1	18.4	22.2	17.9	15.9	21.1	18.2	18.5

### Association between BMI categories, demographics, and disease activity

Patient characteristics and markers of disease activity with regard to the BMI categories are presented in Table 3. In all cohorts, overweight and obese patients were on average 4 years older compared to patients with normal weight. The age at the onset of RA differed, being earlier in underweight patients in RABBIT and NDB (−6.1 years RABBIT, *P* < 0.01; −1.5 years NDB, *P* = 0.61) and later in overweight and obese patients (+3 years CAPEA, *P* < 0.001; +4.3 years RABBIT, *P* < 0.01; +3.4 years NDB, *P* < 0.001) compared to normal-weight patients. This result was confirmed with a separate analysis of 2264 patients with a disease duration <6 months in the entire National Database: these early RA patients (mean disease duration 3.0 months) also showed a later disease onset in overweight/obese compared to normal-weight status (+6 years, *P* < 0.001).

**Table 3:** Clinical characteristics by BMI categories

	CAPEA				RABBIT				NDB			
BMI (kg/m^2^)	<18.5	18.5 to <25	25 to <30	≥30	<18.5	18.5 to <25	25 to <30	≥30	<18.5	18.5 to <25	25 to <30	≥30
Age (years), mean (SD)	58.8 (14.9)	54.2 (15.6)	58.2 (13.6)	57.8 (12.6)	49.8 (15.8)	54.3 (13.7)	58.1 (11.6)	57 (10.9)	59.9 (16.6)	59.7 (15.0)	63.4 (12.6)	61.5 (12.2)
Age at disease onset (years), mean	58.6	53.9	58.0	57.5	37.7	43.9	48.2	48.3	44,4	45,9	50,1	48,1
Disease duration (years), mean (SD)	0.2	0.3	0.2	0.3	12 (9.9)	10.4 (9.3)	9.9 (9.1)	8.8 (8.5)	15.5 (11.2)	13.8 (10.4)	13.3 (10.8)	13.4 (10.5)
Education, high (%)	40.0	21.4	16.4	13.3	22.4	21.7	14.3	11.8	28.3	27.9	18.6	15.7
Smoking, current (%)	63.6	36.3	32.3	27.6	30	24.2	20.7	18	35.7	22.4	19.8	13.8
Smoking, former (%)	9.1	23.9	28.0	39.1	12.9	20.9	25.4	28.7	16.7	25.1	30.9	36.3
RF positive (%)	45.5	55.4	49.8	46.9	73.3	74.7	71.6	66.4	72.2	72.4	69.9	72.3
DAS28, mean (SD)	5.9 (0.7)	4.9 (1.3)	5.1 (1.3)	5.2 (1.2)	5.2 (1.3)	5.1 (1.4)	5.2 (1.4)	5.3 (1.3)	3.0 (1.3)	3.0 (1.2)	3.1 (1.2)	3.4 (1.2)
SJC, mean (SD)	9.2 (5.8)	6.4 (5.5)	6.7 (5.7)	6.2 (5.1)	7.1 (5.5)	6.8 (5.6)	6.6 (5.6)	6.2 (5.5)	1.5 (2.4)	1.4 (2.6)	1.3 (2.7)	1.4 (3.1)
TJC, mean (SD)	10.5 (5.2)	9.7 (6.1)	10.4 (6.7)	10.7 (6.5)	8.7 (6.9)	8.7 (6.9)	9 (7)	9.4 (7.1)	1.7 (3.3)	2.0 (3.7)	1.9 (3.6)	2.3 (3.7)
PGA, mean (SD)	6.2 (2.3)	5.2 (2.3)	5.4 (2.3)	5.7 (2.1)	5.9 (2.2)	5.8 (2.1)	6 (2.1)	6.2 (2.1)	4.1 (2.7)	3.8 (2.2)	4.1 (2.2)	4.6 (2.2)
ESR, mean (mm/h)	42.8 (28.3)	31.5 (23.8)	31.9 (23.1)	35.7 (24.0)	32.6 (25.3)	30.2 (23.3)	31.5 (23.1)	32.2 (21.8)	18.2 (16.6)	18.0 (15.7)	19.1 (16.4)	23.1 (17.6)
CRP, mean (mg/l)	19.4 (34.3)	19.0 (34.5)	19.8 (26.5)	20.6 (29.2)	20.6 (29.9)	18.4 (27.9)	18.3 (26.1)	17.4 (24.8)	7.2 (9.8)	9.3 (65.2)	9.9 (50.8)	10.4 (17.9)
FFbH (0–100), mean (SD)	69.9 (17.8)	78.9 (19.0)	74.2 (21.5)	71.2 (21.0)	62.5 (24.7)	66.6 (22.9)	62.9 (23.2)	58.4 (23.1)	74.8 (25.5)	77.2 (23.1)	72.6 (24.0)	67.9 (24.4)
No. of comorbidities, mean (SD)	0.4 (0.5)	0.6 (1.0)	1.0 (1.3)	1.5 (1.6)	1.2 (1.6)	1.2 (1.5)	1.6 (1.7)	2.0 (1.7)	2.7 (2.3)	2.3 (2.1)	2.7 (2.2)	2.8 (2.2)
Prednisone equivalent in mg/day, mean (SD)*	NA	NA	NA	NA	5.3 (4.8)	5.1 (5.5)	5.1 (5.5)	5.3 (7.8)	5.9 (4.0)	4.8 (3.4)	4.8 (3.0)	5.2 (3.8)

*Average daily dose in the last 6 months. In CAPEA, prior treatment with prednisone was not applicable (NA)

*BMI* body mass index, *ESR* erythrocyte sedimentation rate, *DAS28* Disease Activity Score, *FFbH* Hannover Functional Assessment, *PGA* Patient Global Assessment, *SJC* swollen joint count, *TJC* tender joint count, *RF* rheumatoid factor, *SD* standard deviation

In all cohorts, a high educational level was strongly under-represented in obese patients. Current smoking was negatively and former smoking was positively associated with the BMI. Only in RABBIT and CAPEA were obese patients less often seropositive. In all cohorts, the DAS28 and ESR were increased in obese patients, whereas SJCs were not; CRP was only slightly increased in CAPEA and the NDB. The doses of glucocorticoids (average over last 6 months) were not associated with the BMI in the NDB (*P* = 0.55) and RABBIT (*P* = 0.89). Underweight patients presented with an overall high disease activity in CAPEA and higher SJCs and laboratory markers in RABBIT. Compared to normal weight, functional capacity measured by FFbH was lower in underweight and decreased with rising BMI in all cohorts. In a separate analysis of males and females, sex-specific differences in the relation between BMI and ESR, and CRP and FFbH were observed (Additional files 1: Table S1, 2: Table S2 and 3: Table S3).

**Fig. 2:**
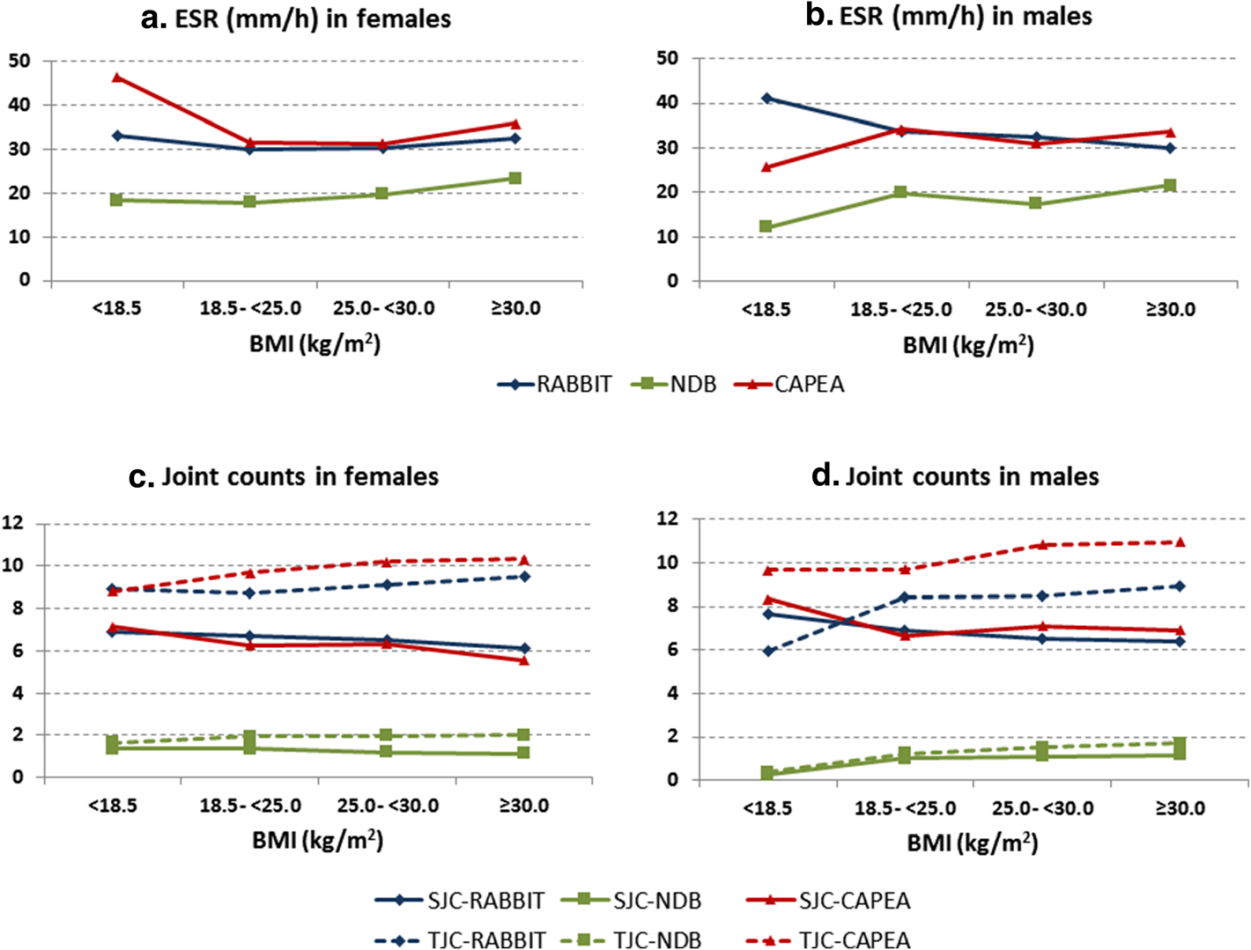
Association between BMI categories and markers of disease activity. Results from generalized linear models for **a** ESR in females, **b** ESR in males, **c** joint counts in females and **d** joint counts in males, adjusted for age, disease duration, glucocorticoid dose (except for CAPEA), and ESR (in the joint count analysis). *BMI* body mass index, *ESR* erythrocyte sedimentation rate, *SJC* swollen joint count, *TJC* tender joint count

To further evaluate the observed differences regarding the association between BMI and the single components of the DAS28, linear models with separate analyses for males and females were performed. Age, disease duration, and glucocorticoid dose (only NDB and RABBIT) were included for adjustment. In females, ESR values were higher in underweight and in obese compared to normal-weight patients in all cohorts. In contrast, in males, ESR values were lower in underweight patients of CAPEA and the NDB and in obese patients of RABBIT compared to normal-weight status. In all cohorts, but especially in RABBIT and in CAPEA, there was an increasing disparity between TJCs and SJCs in higher BMI categories (Fig. 2).

## Discussion

The analysis of three arthritis cohorts within the collaborative METARTHROS network enabled comparison of the BMI distribution in RA patients and the general population. Compared to the reference data, a higher prevalence of obesity was observed in all RA cohorts. Since the reference sample does not exclude persons with RA and other inflammatory diseases, the differences compared to healthy persons is rather underestimated. The prevalence rate of approximately 24 % is higher than that reported from former RA cohorts [12, 13], but similar to current data from the UK [14, 15]. In contrast to the general population, where obesity is equally frequent in females and males, in all RA cohorts it was more frequent in females. The predominance of obesity in female RA patients has already been reported from the Rochester cohort [4]. Reasons for this disparity remain unclear and cannot be explained with the current data. Besides female sex, former smoking and a lower educational level were associated with higher BMI categories. Current smoking was present in the majority of patients with a low BMI but in the minority of obese patients. In addition, obese patients were less frequently seropositive in two of the present cohorts. In other cohorts, seronegative RA has already been reported to be associated with a higher BMI [6]. In accordance with the negative association with smoking, both factors can lead to the reported link of obesity and less structural damage [12, 16], independent of the disease activity or functional status. Increased joint pain and worse functional capacity in obese patients, both of which are observed in the present study, can also result from obesity and its referring comorbid conditions rather than from RA severity [6].

RA manifested at a later age in overweight and obese patients. This is supported by recent data from large population-based Swedish health survey where a higher BMI was associated with a reduced risk of future RA in men, but not in women [17]. The higher proportion of obese patients was already present in the early arthritis cohort CAPEA, where the mean symptom duration of arthritis was less than 4 months. It is possible that being overweight or obese is an acquired risk factor that becomes apparent at an older age. We observed increased joint pain and worse functional capacity in obese patients, which was debated by otherauthors to rather result from obesity itself and the referring comorbid conditions than from RA severity [6]. 

The heterogeneous association of various covariates with BMI categories shows that cause and impact of obesity on RA outcomes can hardly be judged. Other aspects linked to obesity, such as physical activity, failed attempts to lose weight, co-medication, and treatment adherence, probably affect clinical RA outcomes.

Regarding the relation between BMI and markers of disease activity, the composite score DAS28 is not an appropriate measure as it masks the different associations of the single components of the DAS28 as well as age- and sex-specific differences in the markers of disease activity [18]. In the present study, the effect of the BMI on inflammatory markers was not very pronounced. However, joint counts do have a substantial influence on DAS28 values and the dissociation between TJCs and SJCs in obese patients may be responsible for underestimating joint activity in obese patients. This distinct behaviour should be recognized when analysing the impact of BMI on treatment response.

Other interactions may be responsible for the effect observed on the ESR in females. Hormones, cytokines, growth factors, and intracellular regulators can modify the course of inflammation [19], and may be differently distributed in females.

### Limitations and strengths

The BMI was used with WHO definitions for underweight, overweight and obesity; however, it does not measure adipose tissue. The body composition might be more precisely represented by waist circumference, dual-energy X-ray absorptiometry, and fat mass indexes using bioelectrical impedance [5, 20]. We selected the BMI as a surrogate for obesity and underweight as it was obtained in all arthritis cohorts and in the reference group. By using the BMI we could compare a very large dataset at different stages of the disease with the general population, and results could be discussed against the background of data from other cohorts using the same BMI categories [2, 4, 14].

## Conclusions

An increased prevalence of obesity was found in all RA cohorts. A delayed onset of RA was observed in overweight and obese patients. Prospective data are needed to analyse BMI development prior to and its influence on RA onset, since obesity could be approached in prevention campaigns. Differences in the age at onset, the clinical presentation, and the presence of rheumatoid factor in normal-weight and obese patients may partly explain the obesity paradox in the structural course of RA. The predominance of obesity in females and the different behaviour of ESR values in relation to BMI in females necessitate further investigation when analysing the impact of obesity on inflammation in RA.

## Abbreviations

BMI, body mass index; CAPEA, Course And Prognosis of Early Arthritis; CRP, C-reactive protein; DAS28, disease activity score; DEAS, Deutscher Alters survey (German Ageing Survey); DMARD, disease-modifying anti-rheumatic drug; ESR, erythrocyte sedimentation rate; FFbH, Funktionsfragebogen Hannover (questionnaire of functional capacity); METARTHROS, Metabolic impact on joint and bone disease network; NDB, national database (of the German Collaborative arthritis centres); RA, rheumatoid arthritis; RABBIT, Rheumatoide Arthritis Beobachtung der Biologika Therapie (German biologics register); SJC, swollen joint count; TJC, tender joint count; WHO, World Health Organization

## Additional files

Additional file 1: Table S1. Clinical characteristics by BMI and sex categories (CAPEA). (DOCX 14 kb) Additional file 2: Table S2. Clinical characteristics by BMI and sex categories (RABBIT). (DOCX 18 kb) Additional file 3: Table S3. Clinical characteristics by BMI and sex categories (NBD). (DOCX 22 kb)
