# Supplementary material for: Body mass index distribution in rheumatoid arthritis: a collaborative analysis from three large German rheumatoid arthritis databases
Source: Arthritis Res Ther. 2016 Jun 23;18:149. doi: 10.1186/s13075-016-1043-9 (PMC4918111; doi:10.1186/s13075-016-1043-9)
Supplement: Additional file 1: Table S1. — Clinical characteristics by BMI and sex categories (CAPEA). (DOCX 14 kb) [file 13075_2016_1043_MOESM1_ESM.docx]

**Additional file 1: Table S1** Clinical characteristics by BMI categories and sex (CAPEA)

|  | Females | | | | Males | | | |
| --- | --- | --- | --- | --- | --- | --- | --- | --- |
| BMI (kg/m^2^) | < 18.5 | 18.5 - <25 | 25- < 30 | ≥ 30 | < 18.5 | 18.5 - <25 | 25- < 30 | ≥ 30 |
| N | 9 | 260 | 225 | 161 | 2 | 112 | 171 | 82 |
| Age in years, mean (SD) | 60.7 (15.6) | 52.8 (15.7) | 57.8 (14.1) | 57.3 (12.3) | 50.5 (9.2) | 57.3 (15.1) | 58.8 (13) | 58.7 (13) |
| Age at disease onset, mean | 60.4 (15.6) | 52.6 (15.7) | 57.5 (14.1) | 57 (12.4) | 50.3 (9.2) | 57.1 (15.1) | 58.6 (12.9) | 58.5 (13) |
| Disease duration, mean (SD) | 0.3 (0.1) | 0.2 (0.1) | 0.2 (0.1) | 0.2 (0.1) | 0.2 (0) | 0.2 (0.2) | 0.2 (0.1) | 0.2 (0.1) |
| Education, high (%) | 3 (37.5) | 52 (23.3) | 37 (19.8) | 20 (14) | 1 (50) | 17 (17.2) | 19 (12.3) | 8 (11.8) |
| Smoking, current (%) | 5 (55.6) | 84 (32.3) | 63 (28) | 44 (27.3) | 2 (100) | 51 (45.5) | 65 (38) | 23 (28) |
| Smoking, former (%) | 1 (11.1) | 54 (20.8) | 52 (23.1) | 49 (30.4) | 0 (0) | 35 (31.3) | 59 (34.5) | 46 (56.1) |
| RF positive (%) | 5 (55.6) | 113 (43.5) | 116 (51.6) | 86 (53.4) | 1 (50) | 59 (52.7) | 88 (51.5) | 39 (47.6) |
| DAS28, mean (SD) | 6 (0.8) | 4.9 (1.3) | 5 (1.2) | 5.1 (1.2) | 5.6 (0.4) | 5.1 (1.4) | 5.2 (1.4) | 5.3 (1.3) |
| SJC, mean (SD) | 9.4 (6.1) | 6.2 (5.4) | 6.3 (5.4) | 5.7 (4.8) | 8 (5.7) | 7.1 (5.5) | 7.2 (6.1) | 7.1 (5.6) |
| TJC, mean (SD) | 10.7 (5.8) | 9.5 (6.2) | 10.2 (6.6) | 10.5 (6.2) | 9.5 (2.1) | 10 (5.8) | 10.8 (6.8) | 11.1 (7) |
| PGA, mean (SD) | 6.1 (2.6) | 5 (2.3) | 5.4 (2.3) | 5.7 (2.1) | 6.5 (0.7) | 5.5 (2.2) | 5.4 (2.4) | 5.7 (2) |
| ESR, mean (SD) | 47.3 (30.2) | 30.3 (22.9) | 31.7 (23) | 36.3 (23.9) | 25 (4.2) | 34.4 (25.6) | 32.1 (23.4) | 34.4 (24.2) |
| CRP, mean (SD) | 23 (37.8) | 16.2 (33.4) | 15.6 (21.4) | 18.3 (24.7) | 5 (7) | 25.6 (36.2) | 25.5 (31.4) | 25 (36.2) |
| FFbH (0-100), mean (SD) | 71.3 (19.6) | 78.3 (19.3) | 74.9 (20.2) | 70.2 (21.5) | 63.9 (0) | 80 (18.1) | 73.2 (23.1) | 73.3 (19.8) |
| No. of comorbidities, mean (SD) | 0.4 (0.5) | 0.6 (0.9) | 1 (1.4) | 1.5 (1.7) | 0 | 0.7 (1.2) | 1 (1.2) | 1.5 (1.6) |
