# Supplementary material for: Body mass index distribution in rheumatoid arthritis: a collaborative analysis from three large German rheumatoid arthritis databases
Source: Arthritis Res Ther. 2016 Jun 23;18:149. doi: 10.1186/s13075-016-1043-9 (PMC4918111; doi:10.1186/s13075-016-1043-9)
Supplement: Additional file 2: Table S2. — Clinical characteristics by BMI and sex categories (RABBIT). (DOCX 18 kb) [file 13075_2016_1043_MOESM2_ESM.docx]

**Additional file 2: Table S2** Clinical characteristics by BMI categories and sex (RABBIT)

|  | Females | | | | Males | | | |
| --- | --- | --- | --- | --- | --- | --- | --- | --- |
| BMI(kg/m^2^) | < 18.5 | 18.5 - <25 | 25- < 30 | ≥ 30 | < 18.5 | 18.5 - <25 | 25- < 30 | ≥ 30 |
| N | 240 | 3921 | 2969 | 2198 | 23 | 889 | 1325 | 665 |
| Age in years, mean (SD) | 49.8 (15.7) | 53.9 (13.8) | 58.1 (11.8) | 57.1 (11.2) | 49.9 (16.9) | 56.3 (13) | 58.1 (11.1) | 56.8 (10.2) |
| Age at disease onset, mean | 37.5 (16.3) | 43.1 (14.8) | 47.4 (13.8) | 47.8 (12.7) | 40.4 (19.4) | 47.5 (14.3) | 50 (12.4) | 49.7 (11.5) |
| Disease duration, mean (SD) | 12.3 (9.8) | 10.8 (9.4) | 10.7 (9.5) | 9.3 (8.8) | 9.5 (10.6) | 8.8 (8.7) | 8.1 (8) | 7.1 (7.3) |
| Education, high (%) | 56 (23.3) | 861 (22) | 403 (13.6) | 240 (10.9) | 3 (13) | 183 (20.6) | 213 (16.1) | 98 (14.7) |
| Smoking, current (%) | 65 (27.1) | 850 (21.7) | 519 (17.5) | 338 (15.4) | 14 (60.9) | 312 (35.1) | 371 (28) | 178 (26.8) |
| Smoking, former (%) | 32 (13.3) | 758 (19.3) | 569 (19.2) | 533 (24.2) | 2 (8.7) | 247 (27.8) | 521 (39.3) | 289 (43.5) |
| RF positive (%) | 176 (73.6) | 2925 (74.9) | 2089 (70.8) | 1428 (65.7) | 16 (69.6) | 647 (73.4) | 969 (73.6) | 451 (68.6) |
| DAS28, mean (SD) | 5.2 (1.3) | 5.1 (1.3) | 5.2 (1.3) | 5.3 (1.3) | 5.1 (1.3) | 5.2 (1.4) | 5.1 (1.4) | 5.1 (1.4) |
| SJC, mean (SD) | 7 (5.4) | 6.7 (5.6) | 6.6 (5.5) | 6.2 (5.4) | 8 (7.1) | 7.1 (5.9) | 6.7 (5.8) | 6.4 (5.9) |
| TJC, mean (SD) | 9 (6.9) | 8.7 (6.9) | 9.2 (7) | 9.5 (7.1) | 6 (5.8) | 8.6 (7.1) | 8.6 (7.1) | 8.9 (7.2) |
| PGA, mean (SD) | 5.8 (2.2) | 5.8 (2.1) | 6.1 (2.1) | 6.3 (2.1) | 6.5 (1.9) | 5.8 (2.1) | 5.9 (2) | 5.9 (2.1) |
| ESR, mean (SD) | 31.9 (24.8) | 29.4 (22.9) | 30.8 (22.4) | 32.9 (21.6) | 39.5 (29.8) | 33.7 (25) | 33 (24.5) | 30.2 (22.6) |
| CRP, mean (SD) | 19 (28.5) | 16.9 (25.7) | 16.5 (24.3) | 16.8 (22.2) | 37.1 (37.9) | 24.6 (35.3) | 22.3 (29.2) | 19.5 (31.6) |
| FFbH (0-100), mean (SD) | 63.1 (24.6) | 66 (22.9) | 60.1 (23) | 56.1 (22.6) | 56.3 (25.3) | 69.3 (22.8) | 69.2 (22.2) | 65.9 (22.8) |
| No. of comorbidities, mean (SD) | 1.2 (1.6) | 1.2 (1.5) | 1.7 (1.7) | 2 (1.7) | 1.3 (1.7) | 1.5 (1.7) | 1.6 (1.7) | 1.9 (1.7) |
