# Supplementary material for: Body mass index distribution in rheumatoid arthritis: a collaborative analysis from three large German rheumatoid arthritis databases
Source: Arthritis Res Ther. 2016 Jun 23;18:149. doi: 10.1186/s13075-016-1043-9 (PMC4918111; doi:10.1186/s13075-016-1043-9)
Supplement: Additional file 3: Table S3. — Clinical characteristics by BMI and sex categories (NBD). (DOCX 22 kb) [file 13075_2016_1043_MOESM3_ESM.docx]

**Additional file 3: Table S3** Clinical characteristics by BMI categories and sex (NDB)

|  | Females | | | | Males | | | |
| --- | --- | --- | --- | --- | --- | --- | --- | --- |
| BMI(kg/m^2^) | < 18.5 | 18.5 - <25 | 25- < 30 | ≥ 30 | < 18.5 | 18.5 - <25 | 25- < 30 | ≥ 30 |
| N | 50 | 1159 | 832 | 570 | 6 | 257 | 387 | 163 |
| Age in years, mean (SD) | 60.4 (16.7) | 59.2 (15.2) | 63.5 (12.9) | 61.4 (12.5) | 55.3 (17.0) | 62.4 (13.3) | 63.1 (12.0) | 61.8 (11.0) |
| Age at disease onset, mean | 46.1 (17.0) | 44.8 (15.9) | 49.2 (15.1) | 47.5 (14.2) | 30.3 (6.4) | 50.7 (14.8) | 51.8 (14.3) | 49.9 (13.2) |
| Disease duration, mean (SD) | 14.3 (10.4) | 14.3 (10.5) | 14.2 (11.0) | 13.8 (10.7) | 25.0 (14.3) | 11.6 (9.6) | 11.3 (10.1) | 11.9 (9.6) |
| Education, high (%) | 12 (29.3) | 275 (29.1) | 126 (17.9) | 81 (16.0) | 1 (20.0) | 49 (22.6) | 68 (20.1) | 20 (14.6) |
| Smoking, current (%) | 15 (37.5) | 168 (19.8) | 99 (15.4) | 58 (12.8) | 0 | 68 (33.0) | 91 (28.8) | 23 (17.0) |
| Smoking, former (%) | 7 (17.5) | 190 (22.4) | 155 (24.1) | 137 (30.3) | 0 | 74 (35.9) | 141 (44.6) | 76 (56.3) |
| RF positive (%) | 35 (72.9) | 794 (72.6) | 547 (69.2) | 399 (72.9) | 4 (66.7) | 173 (71.5) | 267 (71.2) | 110 (70.1) |
| DAS28, mean (SD) | 3.1 (1.3) | 3.0 (1.2) | 3.2 (1.1) | 3.4 (1.2) | 2.2 (1.2) | 2.8 (1.2) | 2.8 (1.2) | 3.2 (1.3) |
| SJC, mean (SD) | 1.6 (2.4) | 1.4 (2.6) | 1.3 (2.7) | 1.4 (3.0) | 0.5 (0.8) | 1.3 (2.6) | 1.2 (2.7) | 1.4 (3.4) |
| TJC, mean (SD) | 1.8 (3.5) | 2.1 (3.8) | 2.1 (3.8) | 2.4 (3.8) | 0.7 (0.8) | 1.4 (3.1) | 1.5 (3.1) | 1.9 (3.3) |
| PGA, mean (SD) | 4.1 (2.7) | 3.8 (2.2) | 4.3 (2.2) | 4.6 (2.2) | 3.5 (3.0) | 3.6 (2.2) | 3.8 (2.1) | 4.3 (2.2) |
| ESR, mean (SD) | 18.8 (16.2) | 17.6 (15.0) | 20.0 (15.5) | 23.6 (16.9) | 13.3 (20.6) | 20.0 (18.4) | 17.3 (17.8) | 21.6 (19.7) |
| CRP, mean (SD) | 6.7 (9.4) | 6.7 (14.1) | 7.7 (14.4) | 10.5 (18.6) | 13.2 (14.0) | 10.4 (17.6) | 8.7 (13.2) | 10.0 (15.1) |
| FFbH (0-100), mean (SD) | 73.7 (25.8) | 76.0 (23.4) | 69.3 (24.1) | 65.2 (25.0) | 91.7 (14.4) | 82.3 (21.2) | 79.6 (22.2) | 77.0 (19.8) |
| Any Comorbidity (%) | 37 (75.5) | 845 (76.5) | 655 (81.3) | 457 (82.9) | 5 (100) | 168 (68.9) | 297 (78.4) | 132 (82.5) |
| No. of comorbidities, mean (SD) | 2.4 (2.2) | 2.3 (2.1) | 2.8 (2.2) | 2.9 (2.2) | 5.8 (1.9) | 2.2 (2.3) | 2.4 (2.0) | 2.6 (2.2) |
